# Enlarged perivascular spaces and mild behavioral impairment: A cross‐sectional analysis in dementia‐free older adults

**DOI:** 10.1002/alz70856_105336

**Published:** 2026-01-07

**Authors:** Dinithi Mudalige, Dylan X. Guan, Graham A. McLeod, Eric E. Smith, Aravind Ganesh, Zahinoor Ismail

**Affiliations:** ^1^ University of British Columbia, Kelowna, BC, Canada; ^2^ University of Calgary, Calgary, AB, Canada; ^3^ Hotchkiss Brain Institute, University of Calgary, Calgary, AB, Canada; ^4^ University of Toronto, Toronto, ON, Canada; ^5^ Department of Clinical Neurosciences, University of Calgary, Calgary, AB, Canada

## Abstract

**Background:**

Enlarged perivascular spaces (EPVS) and mild behavioral impairment (MBI) are associated with greater dementia risk. We investigated cross‐sectional associations between EPVS burden and MBI presence and symptom severity.

**Methods:**

Participants were dementia‐free older adults in the Comprehensive Assessment of Neurodegeneration and Dementia (COMPASS‐ND) study. EPVS were assessed using a validated visual rating scale applied to T2‐weighted magnetic resonance imaging. MBI was measured using the informant‐reported MBI Checklist (MBI‐C) with a cut‐point of ≥6 for MBI presence. Multivariable logistic and zero‐inflated negative binomial regressions modelled EPVS associations with MBI presence and symptom severity, respectively, adjusting for age, education, and Montreal Cognitive Assessment score, with and EPVS*sex interaction term.

**Results:**

Among 363 participants (52.9% female), every 1‐point rise in total EPVS score was associated with 1.29‐fold greater odds of having MBI (95%CI:1.07–1.54, *p* = 0.007). When examining regional EPVS burden, those with higher centrum semiovale scores had greater odds of MBI (aOR=1.48, 95%CI:1.09–12.02, *p* = 0.01), but this association was not significant for the basal ganglia (aOR=1.39, 95%CI:0.97–1.99, *p* = 0.07) or midbrain (aOR=1.48, 95%CI:0.80–2.76, *p* = 0.21). Total and regional EPVS scores were not associated with MBI symptom severity. None of the associations were moderated by sex.

**Conclusion:**

Older adults with greater global and centrum semiovale EPVS burden are more likely to have MBI. These findings suggest that early microvascular pathology may contribute to later‐life emergent and persistent behavioural symptoms, although longitudinal data are required, along with other indicators of vascular pathology.

